# Brain Abscesses Caused by *Nocardia paucivorans* in a Multiple Myeloma Patient Treated with Lenalidomide and Dexamethasone: a Case Report and Review of Literature

**DOI:** 10.4084/MJHID.2015.011

**Published:** 2015-01-01

**Authors:** Jacopo Monticelli, Roberto Luzzati, Cristina Maurel, Chiara Rosin, Romina Valentinotti, Claudio Farina

**Affiliations:** 1 Unit of Infectious Diseases, University Hospital, Trieste, Italy.; 2 Microbiology and Virology Institute, AO “Papa Giovanni XXIII”, Bergamo, Italy.

## Abstract

We report the first case of multiple brain abscesses caused by *Nocardia paucivorans* in a patient suffering from multiple myeloma on treatment with lenalidomide and dexamethasone. *N. paucivorans* is a recently described species of the genus *Nocardia*, which is supposed to have a heightened neurotropism in cases of disseminated infection. Although nocardiosis itself is an uncommon infectious complication in multiple myeloma so far, nocardial brain abscess should be added to the spectrum of adverse effects due to this novel chemotherapy regimen.

## Case Presentation

A 70-year-old male was transferred to our unit from the intensive care unit of a nearby hospital. He had had sudden onset clonic seizures involving the right hemisoma and the assessment of multiple brain abscesses by computerized tomography (CT) scan. The patient was receiving empirical treatment with metronidazole and cefotaxime. His past medical history included a 2-year-history of IgA myeloma (International Staging System – II). The patient was initially treated with melphalan plus prednisone plus bortezomib (4 cycles) according to MPV protocol. Then, he started treatment with lenalidomide and dexamethasone having completed eleven cycles of such regimen with partial response. During the chemotherapy regimen the patient did not receive a trimethoprim-sulfamethoxazole (TMP-SMX) prophylaxis and, at time of admission, we discontinued the chemotherapy regimen for myeloma. The patient suffered from a 2-month history of subcutaneous abscess of unknown origin on the left shoulder. The lesion was drained, and the microbiological analysis was not conclusive because of mixed bacterial flora. The patient was living in a small rural hamlet. He was a construction worker, but he retired 10 years before the present hospital admission.

**Figure 1:**
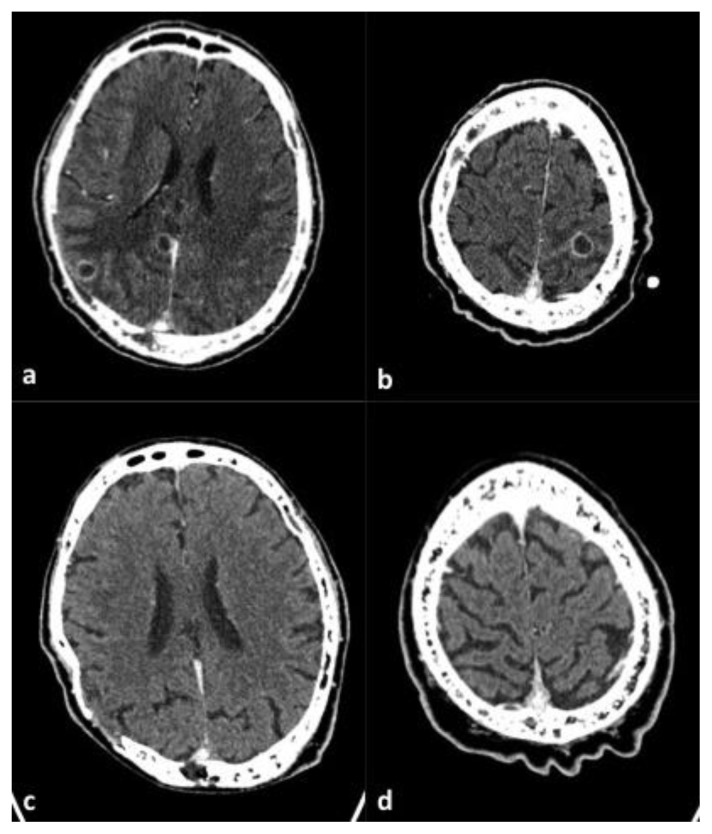
Axial brain CT scan post-contrast medium administration. Images obtained before therapy show two ring-enhancing lesions with abundant peripheral oedema in right parietal lobe (a) and one in left parietal lobe at the vertex (b). Images obtained after therapy (c,d) show the complete resolution of the lesions.

**Figure 2:**
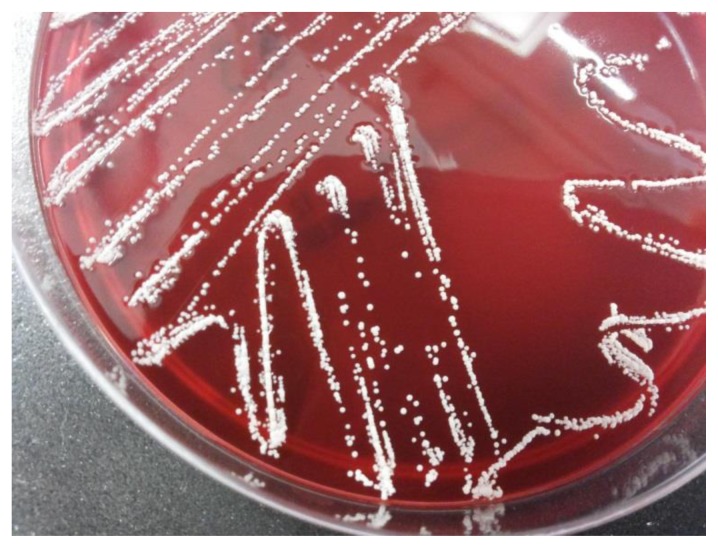
Whitish chalky colonies of *N. paucivorans* yielded on blood agar.

**Figure 3:**
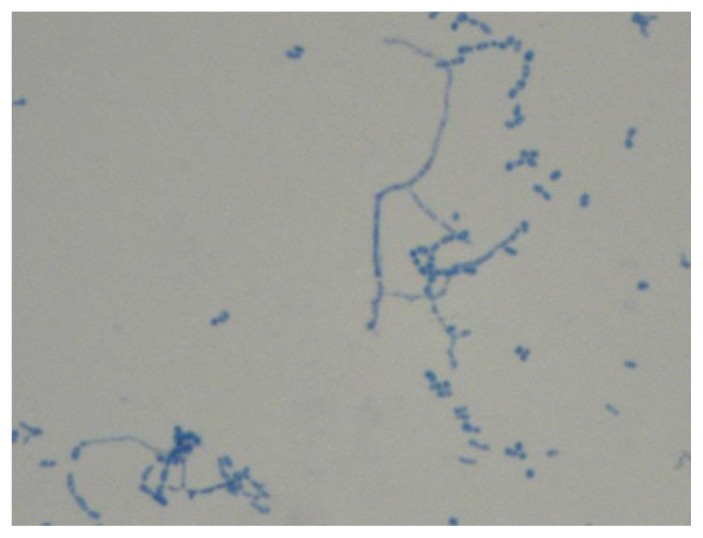
*N. paucivorans* hyphae. Staining by partial acid-fast method according to Kinyoun technique modified for nocardioforms. The vegetative hyphae are well developed with irregular branches.

At hospital presentation, body temperature was 36.5 °C, heart rate was 92/min, blood pressure was 150/90 mmHg and Glasgow Coma Scale was 15/15. The patient was alert, oriented and no abnormal physical findings were shown, except for a painful hypoesthesia in the lateral and anterior sides of the left thigh, in the lateral side of the left leg and, on the dorsal side of the left foot. The subcutaneous abscess on the left shoulder completely resolved with no signs of local inflammation. Laboratory showed the following abnormalities: serum C-reactive protein 96.19 nmol/L (normal values inferior to 71.43 nmol/L), hemoglobin 90 g/L, white blood cells 3.9 × 10^9^/L (51% neutrophil granulocytes). In addition, the analysis of peripheral blood lymphocytes subsets showed CD3+ 742 cells/μL, CD3+CD4+ 420 cells/μL (47%), CD3+CD8+ 241 cells/μL (27%), CD4+/CD8+ ratio 1.70, CD19+ 72 cells/μL. Serum protein electrophoresis showed a relative hypoalbuminemia (55.4%) according to the known monoclonal gammopathy (serum IgA 4490 mg/L, monoclonal component 3 g/L). The chest X-ray revealed a lower right lobe infiltrate associated with bilateral pleural effusion. A brain CT scan (Figure 1) with contrast medium showed 6 focal ring-enhancing lesions of which the largest one had a maximum diameter of 16 mm (left occipital lobe). All the lesions had a minimal perilesional oedema. The right frontal lesions led to a mass effect on the right lateral ventricle. At that time, the patient underwent CT-guided brain biopsy of a parietal lesion. A bioptic sample revealed branching Gram positive nocardioform rods. Then, cultures were incubated aerobically at 35°C for three weeks. All whitish, chalky and wet hay-smelling colonies (Figure 2) yielded on blood agar were stained by partial acid-fast method according to Kinyoun technique modified for nocardioforms (Figure 3).

The suspicion that this strain could belong to *Actinomycetales* genus was based on its failure to clear casein (Biorad, France), on the resistance to lysozime and 5-fluorouracil, and on the enzymatic profile, by the API ZYM system (bioMérieux, France).^1^–^2^ Identification was completed by the evaluation of the absence of urease after growth on Christensen urea agar and incubation at 25°C for three weeks and of the incapacity to clear casein (Biorad, France). Finally, genotypical identification has been performed by amplification and sequencing of a DNA fragment coding a rRNA 16S according with the manufacturer’s (Applied Biosystem, USA) suggestions. Sequences have been compared with the reference ones present in the Gen Bank (NCB) database by the BLAST program. The sequence has been deposited in the GenBank (accession number: AY262324.1). Sequences producing significant alignments permitted to identify the strains as *N. paucivorans* with 100% value of identity with the reference strain.

Minimal Inhibitory Concentrations’ (MIC) testing was performed by the Epsilometer (E test, bioMérieux, France) testing. MICs’ results were the following: amikacin 0.064 μg/mL, amoxicillin plus clavulanic acid (>256 μg/mL), ceftriaxone (0.75 μg/mL), cefepime (6 μg/mL), ciprofloxacin (0.047 μg/mL), clarithromycin (1 μg/mL), gentamicin (8 μg/mL), imipenem (0.19 μg/mL), linezolid (0.125 μg/mL), tobramycin (0.047 μg/mL), trimethoprim plus sulfonamide (0.002 μg/mL). Susceptibility testing was performed on Mueller Hinton Agar (bioMérieux). The results were read after 24 h of incubation at 37°C. Susceptibility testing was revised and interpreted according to CLSI criteria.^3^ In order to exclude concomitant pulmonary nocardiosis, a diagnostic bronchoscopy was done but the microbiological analysis of the bronchoalveolar lavage isolated only commensal microbial flora. As a matter of fact, based on the initial identification and the preliminary antimicrobial susceptibility testing, intravenous TMP-SMX plus meropenem were administered for 24 days. Such antibiotic regimen was switched to oral ciprofloxacin for an overall treatment period of 12 months. The patient is doing well, his painful hypoesthesias have disappeared; no more seizure episodes and adverse effects due to long term antibiotic therapy occurred. The brain CT-scan monitoring showed a progressive reduction of all cerebral lesions (Figure 1).

## Discussion

*Nocardia* (order *Actinomycetales)* is a complex genus of Gram-positive and partially acid- and alcohol-fast bacteria forming irregular branching colonies on agar, including *Nocardia cyriacigeorgica*, *Nocardia farcinica*, *Nocardia brasiliensis* and *Nocardia otitidiscaviarum*, and other *Nocardia* species. *N. paucivorans* is a recently described species, identified by biochemical characteristics and 16S rDNA sequence analysis in bronchial secretions of a patient with chronic lung disease.^4^,^5^

Human nocardiosis is usually recognized as a sporadic, community-acquired infection.^6^ Human nocardiosis affects both immunocompromised and immunocompetent hosts. Within the immunocompromising conditions, nocardiosis seems to affect primarily intravenous drug abusers, patients on systemic corticosteroids, AIDS patients, recipients of solid organ and bone marrow transplants, patients affected by chronic granulomatous diseases and hematologic malignancies. Host resistance to nocardial infection depends on cell-mediated immunity. Infection mainly occurs through direct skin inoculation or inhalation, and possible clinical manifestations are pulmonary nocardiosis, cutaneous and subcutaneous nocardiosis and systemic nocardiosis, including central nervous system (CNS) dissemination.^7^–^8^ CNS nocardiosis can be the result of prior pulmonary infection or can exist on its own. A recent literature review on the nocardiosis reported that CNS involvement can manifest mainly as cerebral abscesses but also as meningitis or both brain abscess and meningitis.^9^ The major species reported among the patients in this literature review were *Nocardia asteroides* (35%), *N. farcinica* (19%), and *N. cyriacigeorgica* (6%), even if *Nocardia* nomenclature dramatically changed in the last years complicating all etiological consideration. Other less common species included *Nocardia transvalensis* (4%), *N. brasilensis* (3.6%), and *N. otitidiscaviarum* (2%) and others. Our patient had CNS nocardiosis due to *N. paucivorans*. The largest series of *N. paucivorans* infections published to date included 33 cases collected in northeast Australia over a 20-years span, with dissemination in at least 33% of cases and CNS infection in 80% of disseminated cases. Therefore, it was hypothesized that *N. paucivorans* had an enhanced neurotropism.^10^–^11^

Nocardial infection in multiple myeloma is quite a sporadic finding, often linked as a complication of bone marrow transplants.^12^ There is limited literature on nocardiosis in multiple myeloma patients that did not undergo bone marrow transplant, and there are still fewer cases of nocardial infections that occurred during novel anti-myeloma treatments. For instance, it was recently reported cases of brain abscesses by *N. cyriacigeorgica* in two patients treated with bortezomib.^13^ A PubMed search, crossing “Nocardia paucivorans” and “myeloma”, revealed no cases, and the *N. paucivorans* infections published so far in English literature did not occur in multiple myeloma patients.^10^–^11^,^13^–^15^ Therefore, to the best of our knowledge, the present case is the first infection due to *N. paucivorans* in a patient affected by multiple myeloma.

Our patient had multiple risk factors for nocardiosis. The myeloma-related immunodeficiency includes mainly B cell dysfunction and cellular immunodeficiency^12^,^16^ with significantly reduced numbers of NK and T cells and impaired T cells function.^17^ Lenalidomide is a synthetic derivative of thalidomide, used in multiple myeloma for its immunomodulatory properties. The molecule induces a IL-2-mediated primary T cell proliferation with a concomitant increase in IFN-γ production along with anti-angiogenic, anti-proliferative, and pro-apoptotic properties.^18^ However, lenalidomide combination therapy has shown to be prone to an increased risk of infections. In fact, a meta-analysis of efficacy and safety of lenalidomide for multiple myeloma showed an increased risk of infections comparing lenalidomide regimen versus placebo (RR = 1.98; 95% CI: 1.50 to 2.62).^19^ Therefore, an antibiotic prophylaxis should be considered as part of the treatment regimen.^19^ Bortezomib induces apoptosis preferentially in rapidly proliferating and neoplastic cells via inhibition of NF-κB activation. However, proteasome inhibition could influence the antigen processing and cytotoxic T-cell response and suppress essential immune functions of human CD4+ T cells. It has been shown that bortezomib therapy could increase the rate of opportunistic infectious complications, mainly within eight cycles of treatment during severe lymphocytopenia treatment-induced periods.^13^,^20^ Corticosteroid therapy is widely associated with nocardial infections. Dexamethasone-based regimens in multiple myeloma increase the rate of bacterial infections (mainly encapsulated bacteria such as *Staphylococcus aureus,* and others including *Enterobacteriaceae*, *Pseudomonas aeruginosa,* mycobacteria)*,* viral and micotic infections.^21^ An observational study of 13 patients collected over a 13-years period in two Spanish institutions showed that the most common risk factor for both pulmonary and disseminated nocardiosis was corticosteroid therapy (64% in the whole group of patients, 45.5% in disseminated nocardiosis patients).^22^ Dose and duration of steroid treatment in nocardiosis patients receiving corticosteroids prior to diagnosis varied widely in that study ranging from prednisone 40 mg/day for 15 days to prednisone 7.5 mg/day for 13 years.

Our patient received for the nocardiosis a combination regimen of TMP-SMX plus meropenem that was switched to a long term oral therapy with ciprofloxacin. Optimal antimicrobial treatment regimens have not been firmly established for nocardiosis, and the management of the disease must be individualized. Immunosuppressed patients and those with CNS disease should receive at least 12 months of antimicrobial therapy with the appropriate clinical monitoring.^7^ In CNS nocardiosis, the antibiotic regimen should include drugs with a favorable CNS penetration, as the ones we used for our patients.

In conclusion, this is the first report of multiple brain abscesses caused by *N. paucivorans* in a patient suffering from multiple myeloma. In this patient the risk factors for a nocardial infection included the hematological disease and chemotherapy. Among the various chemotherapy regimens, we suggest that corticosteroids and lenalidomide had a pivotal role as risk factors for nocardiosis in the present case. Although nocardial infection itself in multiple myeloma is an uncommon infectious complication so far,^13^ nocardial brain abscesses should be added to the spectrum of adverse effect due to the novel antimyeloma drugs. We emphasize the importance in nocardial infections of obtaining the species identification and the strain susceptibility in order to guide the long term antibiotic therapy.

Finally, in our case etiological diagnosis was made possible by culture of brain biopsy. Other than the nocardial infection, the causes of focal brain infections in hematologic malignancies could include for instance neurotoxoplasmosis, zygomycosis, aspergillosis, fusariosis.^23^ Given the risks of misdiagnosis or delayed diagnosis, we highlight the importance of such invasive procedure, as a brain biopsy, to obtain samples for microbiological analysis in all cases of focal brain lesions, especially in the immunocompromised host.

## References

[1] Boiron P, Provost F (1988). In-vitro susceptibility testing of Nocardia spp. and its taxonomic implication. J Antimicrobial Chemother.

[2] Mishra SK, Gordon R, Barnett DA (1980). Identification of nocardiae and actinomycetes of medical importance. J Clin Microbiol.

[3] Clinical and Laboratory Standards Institute (CLSI) (2011). Susceptibility Testing of Mycobacteria, Nocardiae, and Other Aerobic Actinomycetes. CLSI document M24-A2.

[4] Yassin AF, Rainey FA, Burghardt J, Brzezinka H, Mauch M, Schaal KP (2000). Nocardia paucivorans sp. nov. Int J Syst Evol Microbiol.

[5] Wauters G, Avesani V, Charlier J, Janssens M, Veneechoutte M, Delmée M (2005). Distribution of Nocardia species in clinical samples and their routine rapid identification in the laboratory. J Clin Microbiol.

[6] McNeil MM, Brown JM (1994). The medically important aerobic actinomycetes: epidemiology and microbiology. Clin Microbiol Rev.

[7] Wilson JW (2012). Nocardiosis: Updates and Clinical Overview. Mayo Clin Proc.

[8] Corti ME, Villafa-e-Fioti MF (2003). Nocardiosis: a review. Int J Infect Dis.

[9] Anagnostou T, Arvanitis M, Kourkoumpetis TK, Desalermos A, Carneiro HA, Mylonakis E (2014). Nocardiosis of the central nervous system: experience from a general hospital and review of 84 cases from the literature. Medicine (Baltimore).

[10] Gray TJ, Serisier DJ, Gilpin CM, Coulter C, Bowler SJ, McCormack JG (2007). Nocardia paucivorans--a cause of disseminated nocardiosis. J Infect.

[11] Hammoud M, Kraft C, Pulst-Korenberg J, Chenoweth C, Gregg KS (2014). Disseminated Nocardia paucivorans infection in an immunocompetent host. Infection.

[12] Chouci-o C, Goodman SA, Greer JP, Stein RS, Wolff SN, Dummer JS (1996). Nocardial infections in bone marrow transplant recipients. Clin Infect Dis.

[13] Pamukçuoglu M, Emmez H, Tunçcan OG, Oner AY, Cirak MY, Senol E, Sucak GT (2014). Brain abscess caused by Nocardia cyriacigeorgica in two patients with multiple myeloma: novel agents, new spectrum of infections. Hematology.

[14] Eisenblätter M, Disko U, Stoltenburg-Didinger G, Scherübl H, Schaal KP, Roth A, Ignatius R, Zeitz M, Hahn H, Wagner J (2002). Isolation of Nocardia paucivorans from the cerebrospinal fluid of a patient with relapse of cerebral nocardiosis. J Clin Microbiol.

[15] Khan SH, Sanche SE, Robinson CA, Pirouzmand FN (2006). paucivorans infection presenting as a brain abscess. Can J Neurol Sci.

[16] König C, Kleber M, Reinhardt H, Knop S, Wäsch R, Engelhardt M (2014). Incidence, risk factors, and implemented prophylaxis of varicella zoster virus infection, including complicated varicella zoster virus and herpes simplex virus infections, in lenalidomide-treated multiple myeloma patients. Ann Hematol.

[17] Teo SK (2005). Properties of thalidomide and its analogues: implications for anticancer therapy. AAPS J.

[18] Yang B, Yu RL, Chi XH, Lu XC (2013). Lenalidomide treatment for multiple myeloma: systematic review and meta-analysis of randomized controlled trials. PLoS One.

[19] Jung SH, Bae SY, Ahn JS, Kang SJ, Yang DH, Kim YK, Kim HJ, Lee JJ (2013). Lymphocytopenia is associated with an increased risk of severe infections in patients with multiple myeloma treated with bortezomib-based regimens. Int J Hematol.

[20] Nucci M, Anaissie E (2009). Infections in patients with multiple myeloma in the era of high-dose therapy and novel agents. Clin Infect Dis.

[21] Martínez Tomás R, Menéndez Villanueva R, Reyes Calzada S, Santos Durantez M, Vallés Tarazona JM, Modesto Alapont M, Gobernado Serrano M (2007). Pulmonary nocardiosis: risk factors and outcomes. Respirology.

[22] Raturi R, Palacio C, Baluch A, Vargas J, Kenney P, Greene JN (2014). Retrospective analysis of opportunistic brain abscesses in patients with hematologic malignancies. Infect Dis Clin Pract.

